# An Energy-Efficient Multi-Tier Architecture for Fall Detection on Smartphones

**DOI:** 10.3390/s17071487

**Published:** 2017-06-23

**Authors:** M. Amac Guvensan, A. Oguz Kansiz, N. Cihan Camgoz, H. Irem Turkmen, A. Gokhan Yavuz, M. Elif Karsligil

**Affiliations:** 1 Department of Computer Engineering, Yildiz Technical University, 34220 Istanbul, Turkey; irem@ce.yildiz.edu.tr (H.I.T.); gokhan@ce.yildiz.edu.tr (A.G.Y.); elif@ce.yildiz.edu.tr (M.E.K.); 2 IT Department, Garanti Technology, 34212 Istanbul, Turkey; ahmetoguzkansiz@gmail.com; 3 Centre for Vision, Speech and Signal Processing (CVSSP), University of Surrey, Guildford GU2 7XH, UK; n.camgoz@surrey.ac.uk

**Keywords:** activity classification, fall detection, multi-tier architecture, energy efficient smartphone application, simple thresholding, machine learning

## Abstract

Automatic detection of fall events is vital to providing fast medical assistance to the causality, particularly when the injury causes loss of consciousness. Optimization of the energy consumption of mobile applications, especially those which run 24/7 in the background, is essential for longer use of smartphones. In order to improve energy-efficiency without compromising on the fall detection performance, we propose a novel 3-tier architecture that combines simple thresholding methods with machine learning algorithms. The proposed method is implemented on a mobile application, called uSurvive, for Android smartphones. It runs as a background service and monitors the activities of a person in daily life and automatically sends a notification to the appropriate authorities and/or user defined contacts when it detects a fall. The performance of the proposed method was evaluated in terms of fall detection performance and energy consumption. Real life performance tests conducted on two different models of smartphone demonstrate that our 3-tier architecture with feature reduction could save up to 62% of energy compared to machine learning only solutions. In addition to this energy saving, the hybrid method has a 93% of accuracy, which is superior to thresholding methods and better than machine learning only solutions.

## 1. Introduction

In daily life, people could unwillingly be part of some dangerous events resulting in a fall, which may be fatal due to a head injury and/or bleeding. Rapid first aid treatment can potentially reduce the severity of such injuries. However, calling for help could be impossible, especially when the injury results in unconsciousness and injured person is alone in the scene. In this study, we present a novel hybrid method for an assistive mobile application, which exploits the accelerometer sensor of a smartphone for the detection of fall events and informs authorities such as police, ambulance or caregivers to be notified in case of an emergency.

In recent years, there has been a growing interest in fall detection systems. Different fall detection approaches reported so far, are thoroughly examined in different surveys [1,2,3,4,5]. Researchers have exploited several types of sensors, such as accelerometers, cameras, microphones, infrared, floors and pressure sensors to detect fall events. In context-aware systems, visual and audio information was gathered from sensor networks deployed in the environment, especially indoors, whereas accelerometers and gyroscopes were used on the human body and they were introduced as wearable sensors. Both approaches started to lose popularity with the widespread use of smartphones [1]. Advanced features of smartphones make them indispensable to people at any moment. Contrary to external 3-axis accelerometers/gyroscopes and context-aware systems, smartphone-based fall detection applications are non-intrusive and easy to use, even by elderly people, since no beforehand deployment of the sensors is required.

Although the type of sensor(s) to be favored in fall detection applications is quite important, choosing the appropriate sensor data processing technique also plays a crucial role in the success of the system as well as in the energy consumption. In the literature, there are two main data processing techniques to discriminate fall events and fall-like events/daily activities [1,2,3,4,5]: thresholding techniques and machine learning algorithms. Thresholding techniques are advantageous due to their low computational complexity. On the other hand, machine learning methods lead to accurate detection of fall events among the various movement types [1,2]. In this manner, we propose a novel hybrid fall detection method by taking advantages of both approaches. Combining these approaches enabled us to detect fall events in a very energy-efficient way, with high accuracy, low computational cost and low response time. The thresholding phase of the proposed approach, not only eliminates several daily activities, which do not show fall patterns, it also detects severe fall events. Machine learning methods, on the other hand are applied to the remaining ambiguous activities to distinguish fall events from fall-like events. 

The main goal of this study is to discriminate fall events from daily activities with high accuracy in an energy-efficient way. Since accurate detection of fall events is crucial, the parameters of this hybrid system have been adjusted accordingly to increase the sensitivity, specificity and accuracy. As high sensitivity may result in lower specificity ratios, a fall alert handling mechanism has been added to the application to avoid calls caused by misclassified daily activities.

The key contributions of this study could be summarized as follows:

- A new hybrid fall detection approach is introduced in which we take advantages of both thresholding and machine learning algorithms. The thresholding method decreases the computational time, thus saving energy, whereas decision-making based on machine learning algorithms ensures high rates of true positives.
- A novel pre-elimination phase is introduced to save energy, especially in cases where the smartphone retains motionless for extended periods of time, such as when left lying on a desk.
- A mobile application, uSurvive, based on our hybrid fall detection approach has been introduced in this paper. This app runs as a background service and is capable of detecting fall events, and informing the authorities and/or possible caregivers.

The rest of this paper is organized as follows: in Section 2, we briefly discuss the pros and cons of fall detection approaches and elaborate the fall detectors running on smartphones. Section 3 introduces the novel 3-tier architecture of the uSurvive application and describes each tier in detail. We then explain the modules of our mobile application and discuss its advantages in Section 4. In Section 5, we evaluate the proposed architecture and the performance of uSurvive in terms of success rate and energy-efficiency. Finally, we discuss the results and conclude our paper in Section 6 and Section 7.

## 2. Related Work

The fall detection problem has been investigated since the beginning of 1990s. In 1991 Lord and Colvin first proposed to exploit the accelerometer sensor for fall detection [6]. Afterwards, Williams introduced an autonomous belt device, which could detect the impact of the shock on the ground, and a mercury tilt switch to detect the lying position of a human body [5]. However, apart from using accelerometers, there are several different approaches, such as video processing, audio processing, exploitation of gyroscope sensor and processing Wi-Fi signals [7,8] to detect fall events. Compared to visual and acoustic sensors, accelerometers consume less energy and are much easier to integrate into wearable mobility monitoring devices. Several drawbacks of video and audio processing, including their limited area of usage and their high CPU/RAM requirement [9,10,11,12,13,14,15], led the researchers to make more use of wearable sensors [16,17,18]. Moreover, high accuracy rates for fall detection and usability both indoors and outdoors made accelerometer as well as the gyroscope sensors more attractive for fall detection systems.

Several surveys [1,2,3,4,5] about fall detection, published so far, discuss the pros and cons of the proposed approaches from different angles. Each survey has its own categorization and group the available studies based on such as the type of sensors, the applied methods, and/or the intrusiveness of the proposed approach. In the following paragraph, we briefly address these existing surveys.

A comprehensive literature review was completed and published in 2013 [1]. This contemporary study discusses available fall detection systems in terms of the dataset, fall types, performance, sensors and features used for fall detection. In this study, Igual et al. divide fall detectors into two main categories: context-aware systems and wearable devices [1]. The first category includes cameras, floor sensors, infrared sensors, microphones and pressure sensors while the second one includes 3-axis accelerometers either attached to different parts of the body or built-in into a smartphone. Smartphones, being an integral part of many people’s lives, became the center of contemporary studies, since for most people, especially for elderly ones use of a wearable gear is discomforting, thus, they often reject wearing these external sensors. As most smartphones incorporate accelerometers and gyroscopes, the kinematic sensor-based approach [19] could be implemented on them due to their cost effectiveness, portability, robustness, and reliability.

There are also some surveys, which cover existing fall detectors [2,3]. In [2], fall detectors are divided into three groups, including wearable device-based, ambiance sensor-based and camera- based. Perry et al. [3] group the existing studies into three categories based on the measurement techniques used: methods that process acceleration data, methods that measure acceleration combined with other methods, and methods that do not use acceleration. On the other hand, Noury et al. examined available studies based on their detection approaches [4]. According to this study, there are two main approaches. In the first approach, the researchers exploit accelerometer values at the time of impact created by hitting the ground, whereas studies based on the second approach also take the postfall phase into account. Recently, the authors in [20] have compared the thresholding-mechanisms against machine-learning methods [21,22] in terms of classification accuracy. The results in [20] support our proposed approach and show that machine-learning approaches are indispensible for high fall detection rates. On the other hand, [23] has introduced a similar multiphase architecture using wearable sensors to our proposed multi-tier architecture designed for smartphones. However, they do not take energy consumption into consideration in their proposed architecture. Our proposed solution is designed for smartphones and aims at balancing the trade-off between the accuracy and energy efficiency.

In this study, we introduce an energy-efficient, hybrid fall detection model running as a background service on Android smartphones. Thus, we will focus on the fall detection techniques implemented as smartphone applications using different types of sensors including microphones [24], accelerometers, and gyroscopes and elaborate the pros and cons of these techniques. Most of the available studies [25,26] propose thresholding methods. The first smartphone app, PerFallID [27], running on Android G1 was developed in 2010. In this study, the authors exploit a magnetic sensor attached on either chest or waist or placed in a trouser pocket of a person, besides the built-in accelerometer of the smartphone. The experimental results show that, the accuracy of the detection via smartphone accelerometer is 90%, whereas the magnetic accessory improves the success rate by 3%. The energy consumption of PerfallID was examined too, in the same study. Based on the experimental results, the app would last about 33.5 h, if it is left running in the background. On the other hand, one of the first apps published on the Android market, iFall [28], introduces an adaptive thresholding mechanism for fall monitoring and response. Similar to most of the former studies, this app is able to inform social contacts, such as relatives and emergency units, when a fall is detected if not deliberately disabled by the user. In order to reduce the number of false positives, like several other apps, iFall emits a prompt to cancel an alert before it is automatically sent. iFall evaluates both the root-sum-of-squares of the readings from the accelerometer’s three axes and the position of the human body, since people tend to stay motionless for a while after an accident. Another Android application for fall detection was introduced in [29]. This application is based on the principle that accelerometer values indicate zero at the time of a fall. However, the authors did not give any success rate for their threshold-based method.

In [30], the authors model the fall activities with a finite state machine and propose a mechanism which utilizes the accelerometer readings of a smartphone. Only 15 fall events and 15 daily activities were analyzed and the sensitivity and specificity were given as 97 and 100%, respectively. A recent study, [31], labels a daily activity as a fall event if any of the *x*-, *y*-, or *z*-axis accelerometer reading deviates approximately 10 g, within a short period of time, from the average value, calculated from the daily activities of the user.

As to energy efficient fall detection, one of the main problems is the requirement of periodical reading of sensor data [32]. This significantly shortens the battery life of a phone because of the associated energy overhead. Although the sensor itself uses very little power, the problem lies in the need to keep the phone’s main processor and associated high-power components active to achieve sensor sampling at periodical intervals. Thus, the total power consumption for sampling required is orders of magnitude higher than the power actually required for sensing. A recent study [33], clearly shows that the main processors of customary smartphones require more than 1 s to transition between sleep and idle states and vice versa. Hence, floating a smartphone’s main processor between those states in order to achieve energy efficient sampling would not produce eligible sensor data. Thus, in [33], the authors propose the use of a dedicated low-power processor for mobile sensing applications. They underline three different solutions. The first one is to use an existing microcontroller. The second option is to add a dedicated low-power microcontroller to the smartphone. The last one is to insert a low-power core into the smartphone’s main processor. Although the last option is the least costly solution, phone manufacturers would not lean towards it, since it requires changing the main processor. In [34], researchers present Reflex, a suite of compiler and runtime techniques that significantly lower the barrier for developers to leverage such low-processors. Without doubt engaging a low power microcontroller and/or core for energy efficient sampling would be the ultimate solution. However, until smartphones with such specialized hardware for sensing became more customary, we believe that there is still room for more energy efficient operation by engaging hybrid-like solutions as proposed in [23,35] and a hybrid approach with double-thresholding mechanism in this study. The authors in [23] proposed a multiphase architecture but did not take energy consumption into consideration, whereas [35] exploit machine learning algorithms for classifying daily activities (ADLs). Our proposed hybrid approach would also contribute to more energy efficient operation even with specialized hardware.

## 3. Hybrid Model for Fall Detection

**Figure 1:**
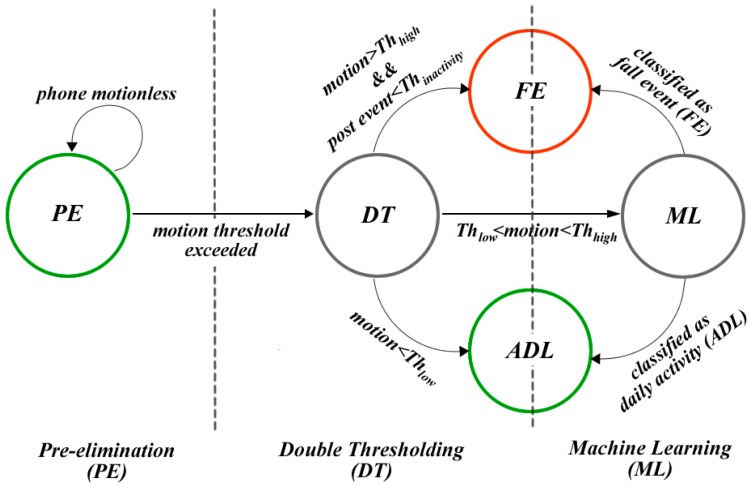
State transitions and inter-tier interactions of the proposed hybrid approach.

The proposed hybrid architecture, that combines simple thresholding methods with machine learning algorithms, allows for energy-efficient operation, which is essential for mobile applications without compromising the performance of fall detection. Our 3-tier architecture, composed of pre-elimination (PE), double thresholding (DT) and machine learning (ML) tiers, shown in Figure 1, accomplishes this goal. The first tier is designed as a filter, which blocks the cases, where the smartphone retains immobile. Thus, unnecessary further processing of sensor data is prevented in this tier. The purpose of the DT tier is to detect harsh falls and physical activities occurring at a slow pace like wiggling and stooping. Finally, the last tier of the fall detection engine utilizes machine learning algorithms to recognize slow falls and fall-like events like sitting, which are difficult to distinguish from actual falls.

### 3.1. Pre-Elimination Tier

People either exhibit a low degree of activity or remain motionless most of the time in their daily lives. Consequently, a smartphone would either be exposed to motions of varying low magnitude or retain motionless as well. The purpose of pre-elimination is to detect such cases and eliminate them before further processing is applied on sensor data. PE tier continuously calculates the difference of successive accelerometer readings for each axis. Let $x_{1}$, $y_{1}$, $z_{1}$ and $x_{2}$, $y_{2}$, $z_{2}$ be two successive accelerometer readings at times $t_{1}$ and $t_{2}$, which are equally spaced in time, corresponding to the system sampling rate. The action is labeled as *motionless* if the following equation is satisfied:

(1) $$\left|x_{1}-x_{2}\right|+\left|y_{1}-y_{2}\right|+\left|z_{1}-z_{2}\right|<Th_{PE},$$

where $Th_{PE}$ refers to a threshold value which is determined based on the analysis of daily activities. This tier is very lightweight and includes only two additions, three subtractions and one comparison. For events that do not meet the Equation (1), the DT tier, as shown in Figure 1, is employed. The DT tier runs relatively more complex instructions in contrast to the PE tier.

### 3.2. Double Thresholding Tier

**Figure 2:**
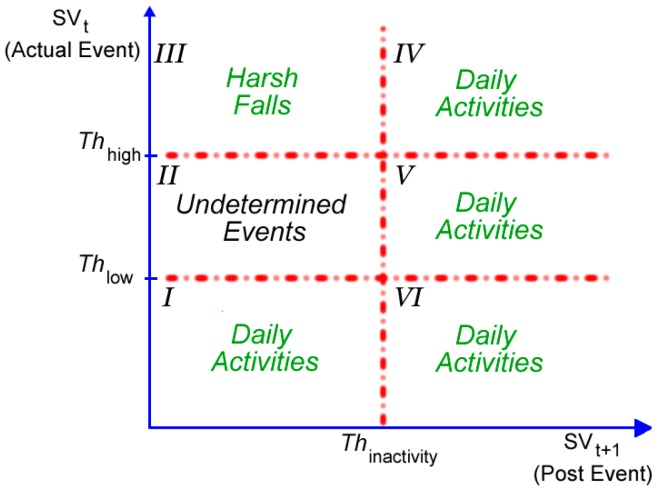
Double thresholding mechanism evaluates both actual and post events performed by the user.

In uSurvive, the detection of harsh falls and physical activities at a slow pace relies on a double thresholding mechanism, which is illustrated in Figure 2. The goal of this tier is to enable fast and accurate decisions for cases involving either a harsh fall or a slow activity without the need for employing machine learning algorithms.

In Figure 2, the actual event is represented by the *y*-axis, whereas the *x*-axis represents the post event. The actual event takes place at a given time t and needs to be classified either as a fall or non-fall event. On the other hand, the event occurring at time t + 1 is the post event and exploited to detect the inactivity state of the victim. The sum vector (${\mathrm{SV}}_{t}$), i.e., signal vector magnitude, of all three axes is calculated using Equation (2), where $x_{t}$,$\;y_{t}$,$\;z_{t}$ correspond to the accelerations along the three axes of the smartphone at a given time t:

(2) $${\mathrm{SV}}_{t}=\sqrt{{x_{t}}^{2}+{y_{t}}^{2}+{z_{t}}^{2}}$$

${\mathrm{SV}}_{t}$ is a common feature also used in other studies to determine a fall event, since a harsh fall always results in a large sum vector. ${\mathrm{SV}}_{t}$ values lower than $Th_{low}$ correspond to daily activities such as walking, wiggling or stooping, which correspond to regions I and VI in Figure 2. All ${\mathrm{SV}}_{t}$ values greater than ${\mathrm{Th}}_{\mathrm{low}}$ require further processing. To this end, we also take the post event at time t + 1 into account and calculate the corresponding a ${\mathrm{SV}}_{t+1}$ value. For a (${\mathrm{SV}}_{t}$, ${\mathrm{SV}}_{t+1}$) pair for which the conditions ${\mathrm{SV}}_{t}$ > $Th_{low}$ and ${\mathrm{SV}}_{t+1}$ > $Th_{inactivity}$ are satisfied, we classify the event as daily activity. Regions IV and V in Figure 2 correspond to this classification. For cases, where ${\mathrm{SV}}_{t+1}$ < $Th_{inactivity}$ and ${\mathrm{SV}}_{t}>Th_{\mathrm{high}}$, we have a harsh fall as indicated by Region III in Figure 2. Region II in Figure 2 corresponds to events, which cannot be classified directly by DT tier using ${\mathrm{SV}}_{t}$ and ${\mathrm{SV}}_{t+1}$ values. ML tier is engaged for the classification of those events. Decision-making by machine learning algorithms involves feature extraction, feature selection and classification of fall and non-fall events.

### 3.3. Machine Learning Tier

The ML tier receives gradual falls and fall-like actions (Region II in Figure 2) such as lying down and sitting that could not be classified by the PE tier or the DT tier. The ML tier is composed of three main steps, namely feature extraction, feature selection and classification. At the first step, time-domain features of events are extracted. Then, feature selection algorithms are employed to determine the most discriminative features. Classification is performed as the last step in order to distinguish fall and non-fall events.

#### 3.3.1. Feature Extraction

In order to extract the set of the most discriminative features that model fall events, feature extraction methods used in fall detection and activity recognition were investigated. These methods can be divided into two groups: time-domain feature extraction methods and frequency-domain feature extraction methods. Computational complexity of transforming raw time-domain accelerometer data into frequency-domain, lead us to prefer time-domain feature extraction methods. According to our observations during dataset collection, a typical fall event lasts at most three seconds. Therefore, accelerometer data acquired at 20 Hz were blocked into frames of three seconds each. A total of 43 features were extracted for each frame [36].

- *Average*: Average acceleration along each axis (3).
- *Standard Deviation*: Standard deviation along each axis (3).
- *Maximum Value*: Maximum value along each axis (3).
- *Average Consecutive Differences*: Average difference of consecutive values along each axis (3).
- *Average Resultant Acceleration*: Average of the square roots of the sum of the values along each axis squared over the frame (1).
- *Binned Distribution*: Fractal distribution along each axis into equal sized bins (30).

Average and Standard Deviation groups contain six basic features, which are also quite discriminative for fall detection. Features in the Average Consecutive Differences group enable utilization of sudden changes in the trajectory of the motion. Average Resultant Acceleration, i.e., signal vector magnitude is calculated using Equation (3):

(3) $$\overset{N}{\underset{i=1}{\sum }}\sqrt{{x_{i}}^{2}+{y_{i}}^{2}+{z_{i}}^{2}}/N$$

where *N* corresponds to the number of accelerometer readings within a frame, which is equal to 60 in our case.

Binned Distribution involves determining what percentage of all values fell within each of the bins. In order to calculate the Binned Distribution feature, we first determined the range along each axis by finding out the respective maximum and minimum values and divided the range into 10 bins of equal size.

#### 3.3.2. Feature Selection

Since a representation that uses too many features increases the computational complexity of machine learning algorithms and may lead to poor prediction accuracy due to the high signal to noise ratio, we exploited feature selection methods to minimize the number of extracted features. 

OneRAttributeEval, RelieFAttributeEval and SVMAttributeEval algorithms were employed and their results were evaluated [37]. The OneRAtrributeEval algorithm evaluates the significance of a feature by using the OneR classifier. OneR learns a one-level decision tree by creating one rule for each feature in the training data. Then, it determines the rule with the smallest error rate as its ‘one rule’ [38]. OneRAtrributeEval selects the feature that gives the minimum error. Relief feature selector was proposed by Kira and Rendell [39]. It evaluates the worth of a feature by repeatedly sampling an instance from the data and then locating its nearest neighbors from the same and opposite classes. The values of the nearest neighbors are then compared to the sampled instance and used to update the relevance scores for each feature [40]. SVMAttributeEval evaluates the significance of a feature by using a linear SVM classifier [37]. Feature ranking is produced by using the square of the weights assigned by the SVM.

The 43 features were ranked using the aforementioned algorithms. To validate the final feature set, we analyzed their variations for 100 fall events and 150 non-fall events, which were acquired by 15 subjects, using three different classification algorithms explained in the following subsection.

#### 3.3.3. Classification

Classification of fall and non-fall events with high accuracy plays a crucial role in ML tier to build a robust model. We considered three different supervised learning approaches for classification: instance-based learning, probabilistic learning and decision tree learning. Constraints on power consumption and CPU utilization made us to choose K-Star [41,42], Naive Bayes [40,43,44] and J48 [45] algorithms which have low computational cost. J48 is the java implementation of the C4.5 algorithm in the Weka [37].

In order to compare the performances of the classification algorithms, 43 time domain features were reduced to 20, 15, 10 and 5 dimensions by using OneRAttributeEval, ReliefAttributeEval and SVMAttributeEval algorithms. Since misclassified falls are much more costly than misclassified daily activities, we compared *F-measure* values of models generated by the combination of classification algorithms and feature selection methods in order to determine the final configuration of our hybrid model. *F-measure* value was obtained according to Equation (4) where true positive (TP), false positive (FP) and false negative (FN) indicate the number of true positive samples, false positive samples and false negative samples respectively:

(4) F-measure=2×TP/(2×TP+FP+FN)

Weka software was used for performing the tests. 10-fold cross-validations were carried out to obtain the success rate of each model. Each fold included the same total number of fall and non-fall events from each subject so that a fair comparison has been carried out. Table 1, Table 2 and Table 3 list the *F-measure* values calculated for the combinations of the classification algorithms and the feature selection methods for the aforementioned number of features. Classification results using features obtained via ReliefAttributeEval, SVMAttributeEval and OneRAttributeEval algorithms are summarized in Table 1, Table 2 and Table 3, respectively.

All models especially the ones generated by using K-Star and J48 algorithms resulted in high *F-measure* values of around 90%. Achieved success rates present the robustness and effectiveness of the feature extraction and classification methods as well as the consistency of the collected dataset. Despite its low complexity, Naïve Bayes falls behind the K-Star and J48. Analyzing the results shows us that, reducing the number of features do not significantly affect the success rate of J48 and K-Star. Since only five features almost achieve the most successful recognition performance of fall events, we implemented J48 with five features selected by the OneRAttributeEval algorithm. Thus, we succeeded in minimizing the complexity of ML tier by removing 38 features.

## 4. uSurvive: Implementation

**Figure 3:**
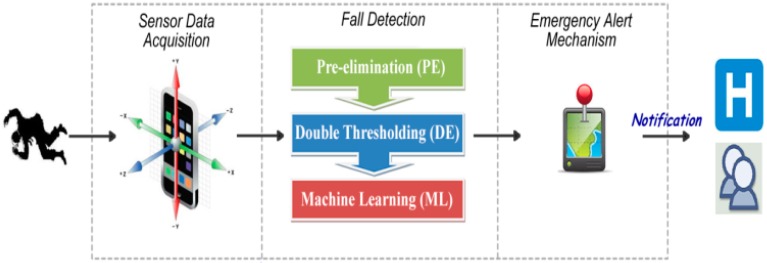
The overview of uSurvive.

uSurvive is composed of three parts: (i) sensor data acquisition (SDA); (ii) fall detection engine (FDE); and (iii) emergency alert mechanism (EAM) as shown in Figure 3. 

The main goal of the application is the detection of fall events, which would require medical attention. While the application is running, accelerometer data are obtained via the sensor data acquisition part and these readings are analyzed in real-time to make sure that no fall event goes undetected. Fall detection part of the application cycles through PE, DT and ML tiers as necessary. Emergency alert mechanism, involves notification of appropriate authorities and/or user defined contacts.

### 4.1. Sensor Data Acquisition

Sensor Data Acquisition (SDA) was implemented using Service and SensorManager APIs of the Android OS. Using these APIs allowed us to run the application in the background. To store the data collected by SDA, a double buffer mechanism was used. While SDA fills in the empty buffer, the other one passed to the FDE. After FDE finishes its processing, the active buffer pointer is switched accordingly. The size of the buffers and the sampling interval are parametric and could be changed during code generation.

### 4.2. Fall Detection Engine

To be able to collect and evaluate accelerometer data concurrently, Fall Detection Engine was implemented as a Runnable (Thread). Whenever a whole buffer of data becomes available for FDE processing, the thread is released via its locking semaphore and the thread locks itself upon its completion.

### 4.3. Emergency Alert Mechanism

EAM is responsible for alerting the caregivers and the authorities whenever a fall event is detected. An acoustic alarm is emitted continuously for 20 s after a fall-like event is reported by the FDE. Besides, a notification message appears on the screen of smartphone. The “Cancel” button needs to be pressed by the victim to deactivate the alarm and prevent the message from being sent. If the victim does not deactivate the alarm within 20 s, the cancel screen is dismissed and a text message is sent to one or more predefined contacts. The message contains the geo-location of the victim.

### 4.4. User Interface

**Figure 4:**
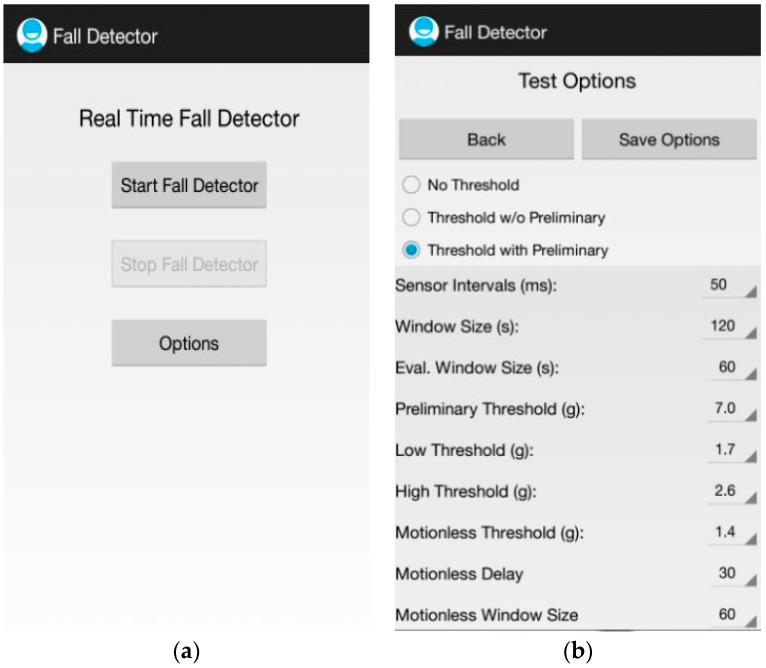
(**a**) The startup screen of uSurvive and (**b**) The setup UI for run-time options.

User interface of uSurvive is composed of two major components: *SDA-UI* and *FDE-UI*:

1. *Sensor Data Acquisition User Interface: SDA-UI* was developed for the collection of the training dataset. 3-axis accelerometer data obtained via *SDA* is stored in a double buffer. “Graphical View” button provides a graphical representation of the buffered data. “Record activity”, “Write to file” and “Delete buffer” buttons are used to extract features from the buffered accelerometer data, to save the extracted features into a file and to empty the buffer manually, respectively.
2. *Fall Detection Engine—UI*: As shown in Figure 4a, *FDE-UI* is composed of three buttons. The first two buttons are for starting and stopping the fall detection engine while the last button provides a way to set up all necessary run-time options of the *FDE* for test scenarios (Figure 4b). When a fall event is detected by uSurvive, an acoustic alarm is emitted and a notification message appears on the smartphone’s screen. If not cancelled within 20 s, a text message is sent to the predefined contacts.

## 5. Performance Evaluation

We evaluated the performance of the proposed method in terms of fall/non-fall events detection accuracy and energy consumption. Then, we present the results in the following subsections.

### 5.1. Hardware and Software Specifications of the Implementation

uSurvive was run on a Samsung Galaxy S3 Mini smartphone [46], running Android 4.1 Jelly Bean [47]. It embeds an accelerometer that acquires 3-axial measurements in the ranges ±2 g/±2 g/±2 g. Accelerometer data was sampled at 20 Hz for 3 s intervals. On the other hand, for the verification of energy consumption results, corresponding tests were also run on a Samsung Galaxy S3 device, which runs Android 4.3.

Thresholding parameters of the DT tier are crucial in the success of the proposed 3-tier hybrid architecture. To achieve user independency in the DT tier, parameters of thresholding and machine learning algorithms were determined using a validation set consisting of 100 fall events and 150 non-fall events. Fifteen subjects were involved in the collection of these events. Subjects performed five different ways of falling: falling while walking, falling while running, falling while standing, falling on knees and falling laterally. They also recorded three different daily activities: walking, running, and standing. During the test scenarios, the subjects were asked to carry the Galaxy S3 Mini in front pocket of their trousers.

**Figure 5:**
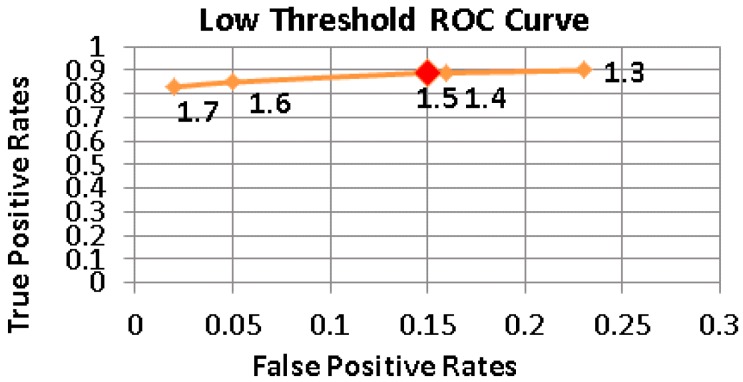
The ROC Curve of Low Threshold values.

**Figure 6:**
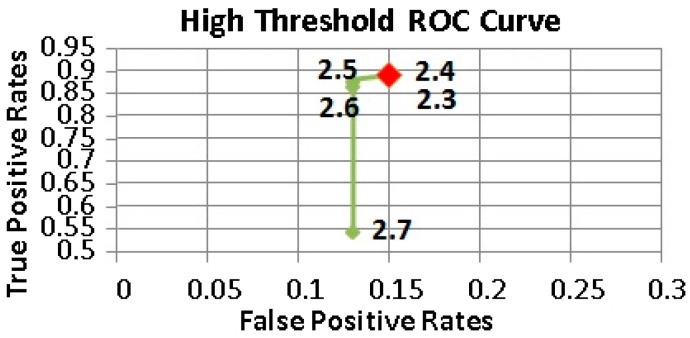
The ROC Curve of High Threshold values.

**Figure 7:**
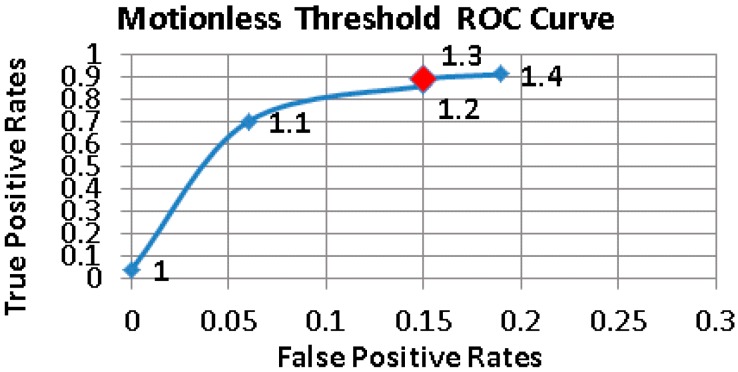
The ROC Curve of Motionless Threshold values.

Figure 5, Figure 6 and Figure 7 show the Receiver operating characteristic (ROC) curves of $Th_{low}$ and $Th_{high}$, $Th_{inactivity}$ respectively. Different combinations of thresholding values were examined to find the appropriate set producing the minimum number of false positives, while maximizing the fall detection rates. The determined threshold values are given in Table 4.

### 5.2. Fall Detection Performance of uSurvive

Fifteen subjects were involved in the performance tests. Subjects imitated five different ways of falling: falling while walking, falling while running, falling while standing, falling on knees and falling laterally. During the test scenarios, the subjects were asked to carry the Galaxy S3 Mini in front pocket of their trousers. In order to make a fair comparison and have a different test dataset, a new set of 175 fall events were first recorded by 15 subjects and then analyzed with three different approaches, only thresholding approach, machine learning only approach and the proposed hybrid method. The obtained results are given in Table 5.

Table 5 clearly shows that the proposed hybrid method outperforms both thresholding and machine learning approaches. The hybrid method improved the success rate up to 4% over the machine learning only approach, which is stated as the most successful approach in the current literature to the best of our knowledge. Sensitivity values seem slightly less than expected. This is to be attributed to the fact that some subjects did not stay motionless after falling down, although they were specifically instructed to do so. Thus, our hybrid approach did not register those cases as fatal falls. After some consideration, we decided to leave them within the dataset.

From the standpoint of the users it is essential for the uSurvive to have a low false positive (FP) rate. As we use 3-s windows for the determination of the event type, approximately 30,000 events are analyzed within a regular working day, i.e., 24 h. An analysis of 24 h out of lab test results shows that only 0.5% of daily activities were misclassified by the proposed hybrid method. Nevertheless, the cancellation option of the generated alerts were adequate to countermeasure this misclassification. On the other hand, machine learning only approach produced 2% misclassifications. The specificity values of the three approaches are given in Table 6.

In addition to specificity and sensitivity, the accuracies of the three approaches are also compared in Table 7. Accuracy is computed by Equation (5) in order to overcome class imbalance:

(5) Accuracy=(Specificity+Sensitivity)/2

We also analyzed the impact of the tiers to show its contribution to the proposed architecture. The daily tests show that pre-elimination tier has eliminated almost 90% of the sampled data while 6% of the sampled data have been eliminated by double thresholding tier. Hence, further computations of sampled data have been prevented and it resulted in energy-savings.

In the former tests, we have trained our system with the samples which are acquired while carrying smartphone in the front pocket of the subject’s trousers. In order to analyze the effects of different carrying types of the smartphones and different body sizes of subjects to the success of the proposed hybrid method, we collected a new dataset consisting of 150 fall events and 900 non-fall events by 10 different subjects including four females and six males. The weights of the subjects ranged from 50 to 95 kg while their heights varied between 155 and 185 cm. During the tests, the smartphone was carried in three different positions: in the front pocket of the subject’s trousers, in the rear pocket of the subject’s trousers and in an inner pocket of the subject’s jacket. The tests involve five different types of falls: backward falling, side falling, forward falling, slow-pace falling and sudden falling while walking, running or standing and six different types of daily activities: walking, running, climbing up and climbing down, sitting down and standing up. Subjects repeated each daily activity for five times. Corresponding confusion matrixes for each carrying type are given in Table 8. The sensitivity of the system when the smartphone is carried in the front pocket of the subject’s trousers, in the rear pocket of the subject’s trousers and in an inner pocket of the subject’s jacket are calculated as 84, 80 and 88% respectively, while the specificities of all three modes are calculated as 99.3%. The results indicate that how the smartphone is carried does not affect the specificity of the system, whereas carrying the smartphone in the inner pocket of jacket positively affects the success of fall detection as it enables more acceleration due to the higher position of the smartphone on the body.

In order to analyze the effect of body sizes of the subjects to the performance of the hybrid method, subjects were separated into three groups according to their weights: Subjects within a range of 50–60, 61–70 and 71–95 kg. Confusion matrixes of each group are given in Table 9.

Sensitivity of the system for three different weight groups 50–60, 61–70 and 71–95 kg are calculated as 81.6, 80 and 91.1%, respectively, while specificity values are calculated as 100, 99.2 and 98.5%. It is observed that while there is no significant difference between the success rates of 50–60 kg group and 61–70 kg group, there is a fair amount of increase in sensitivity and specificity values of 71–95 kg group. This difference can be explained by greater impact values produced while the large-size bodies are hitting the ground.

Comparisons of the proposed hybrid method with only thresholding approach and with machine learning only approach are also performed using asymptotic McNemar’s test in order to assess statistical significance of the results [48]. Confusion matrixes of three approaches are given in Table 10.

The null hypothesis (H0) for the first test suggests that machine learning only approach performs better than the proposed hybrid method whereas the alternative hypothesis (H1) claims the contrary. The result of McNemar’s test indicates rejection of the null hypothesis at the 5% significance level where the *p*-Value of the test is calculated as 2.3 × 10^8^. Comparing the proposed hybrid method with only the thresholding approach by using McNemar’s test results in rejecting the null hypothesis that suggests only thresholding approach performs better than proposed hybrid method at the 5% significance level where *p*-Value of the test is calculated as 6.2 × 10^−3^.

### 5.3. Energy Consumption

Power consumption is one of the most important criteria for mobile application evaluation. Thus, several optimization techniques could be introduced regarding the most energy consuming components of smartphones [49,50]. Although there is no standard way of monitoring the energy consumption of a mobile application running on a smartphone, there are applications which enable the monitoring of the energy consumption of both system components and applications. PowerTutor [51] is one of such applications and to the best of our knowledge, it is the most reliable one. Therefore, we used PowerTutor for the energy measurement tests and to be confident about the obtained results we conducted all the corresponding tests on two separate phones with different models, namely the Galaxy S3 Mini and Galaxy S3.

Before comparing and evaluating the energy consumption of the three approaches—only thresholding approach, machine learning only approach and hybrid method—we investigated the impact of feature reduction on the energy consumption of ML only approach. To this end, uSurvive was configured to run the ML only approach with 43 and five features, respectively, for a duration of 5, 10 and 30 min. The test results, given in Table 11, show that the energy consumption is a linear function of time and feature reduction provided up to 32 and 50% energy savings on the S3 Mini and S3, respectively.

**Figure 8:**
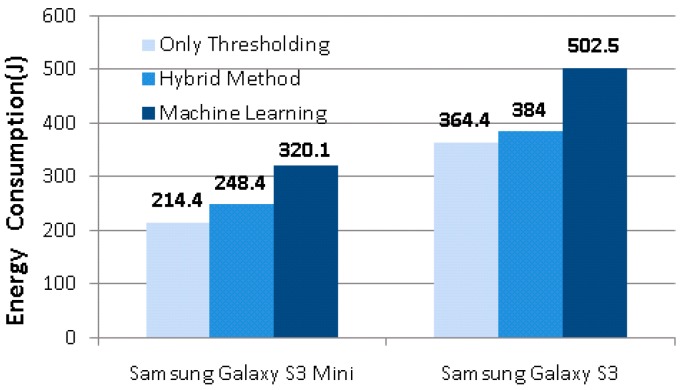
Energy consumption values of three approaches for 24 h.

Next, all the three modes were tested on both smartphones for a duration of 24 h each by a 23-year old computer engineer affiliated with our lab. He spent a total of six days performing the tests. The 24 h period began just after the midnight and ended by the next midnight. Each 24 h period consisted of 7 h of sleep, 8 h of work with the rest involving other personal activities. The test results, given in Figure 8, show that almost 25% of energy could be saved via the hybrid method against the ML only approach. 

We also analyzed the battery remaining power running the system with/without hybrid approach. The results show that running uSurvive with the hybrid approach adds only 2% additional burden to the battery consumption in a daily usage of smartphone.

The PE and DT tiers perform thresholding on the difference of accelerometer readings (Equation (1)) and signal vector magnitude of all three axes (Equations (2) and (3)), respectively. Since only thresholding consists of a single comparison, computational complexity of both tiers is O(n) where n is the number of inputs, which is three in our case for fall detection due to the acceleration values at a given time t of x, y and z axes. On the other hand, the test complexity of the C4.5 algorithm corresponds to the tree depth, which cannot be larger than the number of attributes [52]. Since the system is trained by only five features, the depth of the produced tree offers a reasonable test complexity for a mobile application. However, the computational cost of the feature extraction steps increases the overall complexity of the ML tier. As demonstrated in Figure 8, the energy consumption of the system is directly affected by complexity values of two approaches: only thresholding and machine learning only. However, when the hybrid method is examined, it is observed that PE tier has eliminated almost 90% of the sampled data while DT tier has eliminated 6% of them. The ML tier classified the remaining 4% of the samples. This structure results in very close energy consumption of only thresholding approach and hybrid method and therefore ensures a remarkable energy saving compared to machine learning only solution.

**Figure 9:**
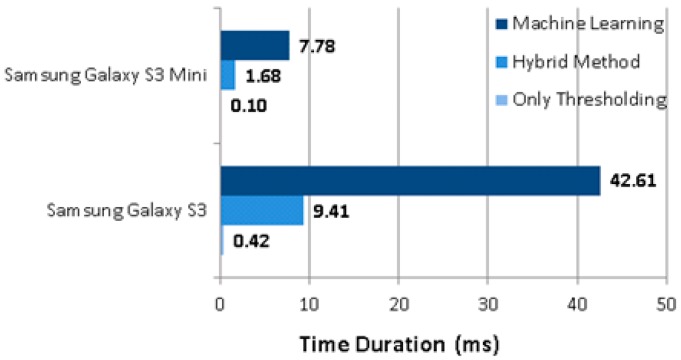
CPU time of the three approaches.

In addition to energy consumption, the computational time required for each approach was explored too. The computational time results of the 100 fall events were averaged for each approach respectively and presented in Figure 9. The results show that the hybrid method runs approximately 4.5 times faster than the ML only approach on both of the smartphones.

**Figure 10:**
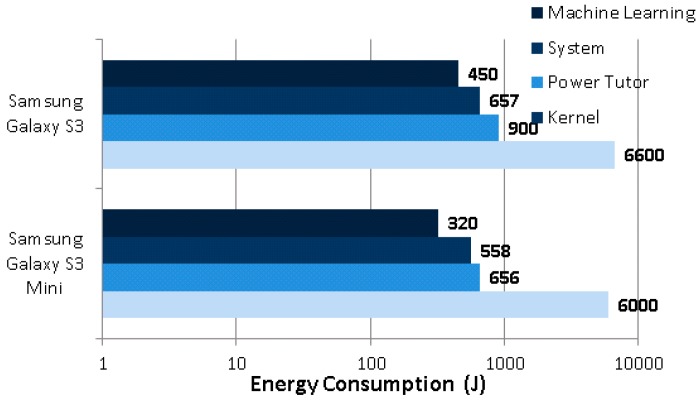
The most energy consuming apps and system components.

In order to emphasize the importance of saving energy for fall detection, we also compared the most energy consuming components during the tests. With the ML only approach, only three other components besides the fall detector come into consideration, kernel, system, and PowerTutor, as given in Figure 10. 

It is evident that on both of the phones, the ML only fall detector consumes nearly as much energy as the Android system itself. Therefore, the aforementioned savings of 25% by our proposed hybrid method is crucial in extending the usage time of the smartphone.

## 6. Discussion

In this study, a novel hybrid model, which reduces power consumption of the system by combining thresholding with machine learning methods, is introduced. Due to its experimental success and low test complexity the C4.5 algorithm is exploited in the ML tier of the proposed method. However, the presented hybrid model offers flexibility for using various machine learning methods in the ML tier. A conventional machine learning algorithm with high computational complexity and accuracy or a lightweight machine learning approach which does not require any feature extraction, batched data or training [53] can be employed in the machine learning tier. In the proposed approach, regarding test results, 96% of the data collected in a regular weekday are determined by the PE and DT tiers. The remaining 4% of the samples are sent to the ML tier and classification is made in this tier. Since thresholding is capable of making a decision by employing a single comparison of raw accelerometer data and 96% of data are evaluated by PE and DT tiers, very close low energy consumption values of only thresholding and hybrid method, as shown in Figure 8, are observed in conducted 24 h tests. These results show that the effect of the computational cost of machine learning algorithm selected for ML tier to the total power consumption of the system is negligible. Thus, a lightweight machine learning algorithm such as Online Perceptron or a fully featured classification algorithm with high-test complexity such as k-Nearest Neighborhood (k-NN) would barely change the energy consumption of the proposed hybrid model.

Fully featured machine learning methods such as SVM, Multi Layer Perceptron and k-NN are proven methods for fall detection [20]. Unfortunately, these methods consume a lot of energy since all acquired samples are processed and feature extraction is required. For this reason, preferring a lightweight machine learning approach then the proposed hybrid model is considered. One of the lightweight algorithms with the lowest query complexity is Online Single Layer Perceptron. For the classification, Online Perceptron uses Linear Combiner. Inputs are multiplied by their respective weights and then summed up. This is followed by a bias correction and a comparison. Therefore, the query complexity of the Online Single Layer Perceptron is O(n) where n is the number of the features, which is not lower than the computational complexity of thresholding solutions [54]. Besides, lightweight machine learning algorithms are successful generally in distinguishing linearly separable classes [55]. Both reasons lead us to present a system that utilizes the proven success of fully featured machine learning approaches in fall detection task while eliminating their disadvantage in terms of energy consumption by employing PE and DT tiers.

Studies that propose multiphase [23,51,56] approaches in order to detect fall events use wearable sensors for data acquisition. We believe that our proposed system can be used by a wide range of audiences since it is developed on a smartphone which is easily accessible to anyone. Its energy efficient architecture would possibly increase the demand to our mobile application. To the best of our knowledge, there are only a few studies which compare the thresholding and machine learning methods [20,23]. However, these studies do not take energy consumption into consideration.

In the current study, we developed a user independent hybrid model. The results have been obtained by using a validation set which is collected by involving 15 subjects. It is expected that configuring the thresholding and machine learning tiers with subject-specific parameters will increase the success rates. As expected, we observed that in our experiments subjects with greater body sizes bring about greater impact values while hitting the ground. Thus, a fair number of falls of subjects with larger body sizes are classified successfully more than fall events of low-to-average weight people. However, regarding total experiments, our test results show that body sizes do not significantly change the fall detection rates of the proposed hybrid approach. Besides, this intrusive approach would bring forth a user dependent system. In order to overcome this problem and increase the accuracy, we believe that deep learning solutions could give a superior fall detection rates without any need to subject-specific parameters.

In our tests, we also realized that the way of falling could affect the fall detection rates. Especially, the very slow falls of low-weight people might be missed. However, the damage risk of these type falls is quite low. We believe that increasing the number of these types of falls in training set would help to the recognition of these fall events.

Another important point is that there are only a few public available datasets [51,57]. One of the most comprehensive databases that include accelerometer, gyroscope and orientation measurements of fall events and daily activities is the MobiFall dataset [58]. In [59], researchers exploit Auto-Regressive model for feature extraction and classify two types of fall events and three types of daily activities by using Support Vector Machine and Neural Network. Comparing two classification techniques, Neural Network provides an overall classification accuracy of 96%, whereas Support Vector Machine results in a classification accuracy of 91.7%. Another study was presented by Vallabh et al. [60]. They implemented a filter rank based system for feature extraction. Five classification methods including Naive Bayes, k-NN, Neural Network, Support Vector Machine, and Least Squares Method were compared to each other. The highest accuracy, calculated as 87.5% is achieved by k-NN algorithm. In order to compare the classification accuracy of the proposed hybrid approach with the studies that use MobiFall dataset, a total of 200 events from MobiFall dataset (100 fall events and 100 daily activities) are classified by the proposed hybrid approach. The average accuracy rates of fall events and daily activities are calculated as 92% and 90% respectively. Regarding these success rates we can safely claim that our hybrid method outperforms the latest studies using the MobiFall dataset in an energy efficient manner.

Our next goal is to publish the collected datasets totally consisting of 425 fall events including five different fall types, three different ways of carrying the mobile phone and 1200 daily activities collected from 25 subjects with a reasonable range of age and body sizes. We also plan to extend our database by including subjects with ages older than 50 to improve the sensitivity. Another improvement could be made for position/orientation problem. During real-life tests mobile phones were carried in front/rear pocket of subject’s trousers and a jacket pocket of the subject. Our test results show that the position of the mobile phone on the user body slightly affects the detection of fall events. We observe that carrying smartphone in the upper part of a body has a positive effect on the detection rate, as the fall event takes longer time than with the lower part of a body. We believe that since changes in orientation of smartphone affect the evaluation, building up the system by taking different transport modes of the mobile phone into account such as carrying in a backpack/handbag or training the system with orientation-independent features will definitely increase the usability of the application.

We also believe that in the near future smartphones would be replaced by smartwatches especially to track people activities. Since from time-to-time we leave our phones on the table, desk, couch, bed, car, bag, etc., smartwatches are the perhaps the most appropriate candidates for analyzing daily activities of people. Therefore, as a future work we are planning to implement this hybrid approach on smartwatches.

## 7. Conclusions

Distinguishing falls from daily activities by using mobile devices is a challenging problem and has been the subject of many studies published in recent years [23]. Developing a successful mobile fall detection system is based on two key factors: energy efficiency and high fall detection accuracy. As stated in [23], in this study, we implemented an out of lab environment and refined our fall detection system for continuous monitoring. Our proposed 3-tier architecture could overcome the energy efficiency problem in fall detection systems designed for smartphones without sacrificing the performance of detection of fall events. There are two main contribution of the proposed 3-tier architecture to the literature:

- Up to 25% of energy was saved by combined use of Pre-Elimination and Double Thresholding tiers, which are capable of detecting smartphone’s immobility, harsh falls and slow pace physical activities.
- The computational complexity in the ML tier has been decreased by selecting the most discriminative features. Reducing the number of features improved the fall detection accuracy while reducing the energy consumption up to 50%.

Thanks to the proposed hybrid approach, we achieved substantial energy savings. As of now, we are working on the implementation of system routines in a native environment instead of the Java environment in order to further decrease the energy consumption of uSurvive.

## Figures and Tables

**Table 1:** *F-measure* values using features obtained via ReliefAttributeEval algorithm.

Number of Features	*F-Measure* Values		
	Naive Bayes	K-Star	J48
43	0.758	0.790	0.875
20	0.788	0.893	0.867
15	0.794	0.888	0.909
10	0.811	0.914	0.912
5	0.807	0.891	0.871

**Table 2:** *F-measure* values using features obtained via SvmAttributeEval algorithm.

Number of Features	*F-Measure* Values		
	Naive Bayes	K-Star	J48
43	0.758	0.79	0.875
20	0.809	0.854	0.899
15	0.8	0.866	0.892
10	0.787	0.896	0.904
5	0.827	0.902	0.889

**Table 3:** *F-measure* values using features obtained via OneRAttributeEval algorithm.

Number of Features	*F-measure* Values		
	Naive Bayes	K-Star	J48
43	0.758	0.790	0.875
20	0.772	0.899	0.896
15	0.779	0.893	0.906
10	0.795	0.892	0.877
5	0.826	0.909	0.902

**Table 4:** Threshold values used in the pre-elimination and double thresholding tiers.

**Pre-Elimination**	
$Th_{PE}$	7
**Double Thresholding**	
$Th_{low}$	1.5 g
$Th_{high}$	2.4 g
$Th_{inactivity}$	1.3 g

**Table 5:** Sensitivity values of the three different approaches.

System Configuration	Sensitivity
Only Thresholding	77%
Machine Learning	82%
Hybrid Method	86%

**Table 6:** Specificity values of the three different approaches.

System Configuration	Specificity
Only Thresholding	99.8%
Machine Learning	98%
Hybrid Method	99.5%

**Table 7:** Accuracy of the three different approaches.

System Configuration	Accuracy
Only Thresholding	88.4%
Machine Learning	90%
Hybrid Method	92.75%

**Table 8:** Confusion matrixes for three carrying types: In front pocket (Fp) of the subject’s trousers, in rear pocket (Rp) of the subject’s trousers and in inner pocket (Ip) of the subject’s jacket.

Predicted							
		Fp of the Trouser		Rp of the Trouser		Ip of Jacket	
Actual		**Fall**	**ADLs**	**Fall**	**ADLs**	**Fall**	**ADLs**
	Fall	42	8	40	10	44	6
	ADLs	2	298	2	298	2	298

**Table 9:** Confusion matrixes of the subjects within a range of 50–60, 61–70 and 71–95 kg.

Predicted							
		50–60 kg (4 Person)		61–70 kg (3 Person)		71–95 kg (3 Person)	
Actual		**Fall**	**ADLs**	**Fall**	**ADLs**	**Fall**	**ADLs**
	Fall	49	11	36	9	41	4
	ADLs	0	360	2	268	4	266

**Table 10:** Confusion matrixes for three approaches: the proposed hybrid method, only thresholding approach and machine learning only approach.

Predicted							
		Hybrid Method		Machine Learning Only		Only Thresholding	
Actual		**Fall**	**ADLs**	**Fall**	**ADLs**	**Fall**	**ADLs**
	Fall	126	24	121	29	114	36
	ADLs	6	894	36	864	4	896

**Table 11:** The impact of feature reduction on energy consumption values.

		Feature Reduction		
	Time (min)	Energy Consumption w/5 Features (J)	Energy Consumption w/43 Features (J)	Energy Saving (%)
Galaxy S3 Mini	5	1.1	1.6	31.25
	10	2.2	3.2	31.25
	30	7	9.4	25.5
Galaxy S3	5	1.4	2.4	41.66
	10	3.1	5.9	47.46
	30	9	18	50
